# *KIR*/*HLA* Pleiotropism: Protection against Both HIV and Opportunistic Infections

**DOI:** 10.1371/journal.ppat.0020079

**Published:** 2006-08-18

**Authors:** Ying Qi, Maureen P Martin, Xiaojiang Gao, Lisa Jacobson, James J Goedert, Susan Buchbinder, Gregory D Kirk, Stephen J O'Brien, John Trowsdale, Mary Carrington

**Affiliations:** 1 Laboratory of Genomic Diversity, SAIC-Frederick, Inc., National Cancer Institute, Frederick, Maryland, United States of America; 2 Department of Epidemiology, Johns Hopkins University Bloomberg School of Public Health, Baltimore, Maryland, United States of America; 3 Division of Cancer Epidemiology and Genetics, National Cancer Institute, Bethesda, Maryland, United States of America; 4 San Francisco Department of Public Health, San Francisco, California, United States of America; 5 Laboratory of Genomic Diversity, National Cancer Institute, Frederick, Maryland, United States of America; 6 Department of Pathology, University of Cambridge, Cambridge, United Kingdom; National Institutes of Health, United States of America

## Abstract

The compound genotype *KIR3DS1/HLA-B Bw4-80I,* which presumably favors natural killer cell activation, has been implicated in protection against HIV disease. We show that this genotype confers dual protection over the course of HIV disease; early direct containment of HIV viral load, and late specific defense against opportunistic infections, but not AIDS-related malignancies. The double protection of *KIR3DS1/Bw4-80I* in an etiologically complex disease such as AIDS, along with the disease specificity of its effects is conceptually novel and underscores the intricacy of host immunogenetics against HIV/AIDS.

## Synopsis

Natural killer (NK) cells are part of the innate immune response which provides the first line of defense against viral infections such as HIV by production of cytokines and direct killing of infected cells. NK cells possess a variety of inhibitory and activating receptors that upon binding to their HLA class I ligands on target cells, regulate activation and inhibition of NK cell responses. A protective effect of the specific combination of the activating receptor *KIR3DS1* with *HLA-Bw4* alleles that have isoleucine at position 80 *(HLA-B Bw4-80I)* against AIDS progression was reported previously. Based on this genetic association, KIR3DS1 on NK cells was proposed to bind to HLA-B Bw4-80I on HIV-1 infected target cells, thereby signaling the NK cell to kill the target. Here we present data showing that this compound genotype also confers protection against the development of AIDS defining opportunistic infections. Interestingly, no protection against the development of AIDS defining malignancies was observed. The double protection of this compound genotype in AIDS, along with the specificity of its effects is a novel finding and underscores the complex role of host immunogenetics against HIV/AIDS.

## Introduction

Natural killer (NK) cells are central components of the innate immune response, providing early defense against viral infections and tumor cells by production of cytokines and direct cytotoxicity [1,2]. Regulation of their activity is under the control of a range of activating and inhibitory receptors that work in concert to identify and destroy aberrant target cells, while recognizing and sparing unblemished self [3,4]. The group of killer immunoglobulin-like receptors (KIR) on NK cells, which contains allotypes that are either activating or inhibitory, participate in the complex regulation of NK cell responses through recognition of specific human leukocyte antigen (HLA) class I molecules on target cells [3]. *KIR* and *HLA* loci are both highly polymorphic and they map to distinct human chromosomes (Chromosomes 19 and 6, respectively), and therefore, they segregate independently. Both the KIR receptor and its specific HLA ligand must be present in order to regulate NK cell activity, such that one without the other is functionally inert. The specific combination of the activating *KIR* allele *KIR3DS1* with *HLA-B* alleles that encode molecules having isoleucine at position 80 *(HLA-B Bw4-80I)* was previously observed to exert a protective effect against AIDS progression after HIV infection based upon a genetic association analysis of AIDS cohorts [5]. We proposed that KIR3DS1 might bind HLA-B Bw4-80I allotypes on target cells, thereby signaling the NK cell to kill the HIV infected target, although direct evidence for a KIR3DS1: HLA-B Bw4-80I interaction has not been reported. The synergistic protection of *KIR3DS1 + HLA-B Bw4-80I* (termed “*KIR3DS1/Bw4-80I”* hereafter) was observed against progression to CD4 T cell depletion and development of AIDS-defining illnesses collectively. Thus, *KIR3DS1/Bw4-80I* may confer protection against HIV directly by killing HIV-infected target cells, and/or indirectly by preventing/delaying onset of specific AIDS-defining illnesses.

## Results/Discussion

AIDS-defining illnesses include two basic types of diseases, opportunistic infections and certain malignancies. Given the protective effect of *KIR3DS1/Bw4-80I* on progression to CD4 T cell decline and AIDS in general, we tested the specificity of this genotype in defense against individual AIDS-defining illnesses and against HIV directly. The most common AIDS outcomes in our cohorts include two malignancies, Kaposi sarcoma (KS) and AIDS lymphoma, and three diseases caused by opportunistic infections (OI): pneumocystis carinii pneumonia (PCP), cytomegalovirus retinitis (CMVR), and mycobacterium avium complex (MAC). The other OI observed in our cohort of patients were grouped together in this study because their individual frequencies were too low to consider individually (termed “other OI”; see Table 1 footnote). The combined group of all OI and combined AIDS-related malignancies occurred at similarly advanced stages of HIV-related immune suppression (mean time to CD4 <200: 5.8 vs. 6.5 y; *p* >0.1).

**Table 1:**
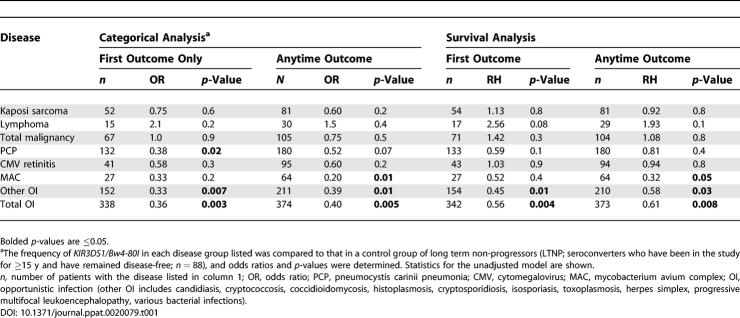
Effect of *KIR3DS1/Bw4-80I* across Distinct Disease Outcomes in HIV-1 Infection

Disease-free survival and categorical analyses were performed on a group of 1,184 study participants whose date of HIV seroconversion was known within a 6-mo period on average (seroconverters). All clinical data used in this study was collected before the advent of highly active antiretroviral therapy (HAART) in order to avoid confounding of our results by efficacious antiretroviral therapy. In categorical analyses, the frequency of *KIR3DS1/Bw4-80I* in each of six individual disease groups (KS, lymphoma, PCP, CMVR, MAC, other OI) and combinations of these groups (malignancies combined and all OI combined) were compared to the frequency of this compound genotype in a control group of 88 HIV+ long-term nonprogressors (LTNP) who had participated in their respective studies for at least 15 y and had never presented with disease symptoms. Since the control group was consistent for all categorical analyses, effects of *KIR3DS1/Bw4-80I* across distinct disease outcomes could be compared directly. In the primary analysis, individuals were included in a disease group only if that disease was the first AIDS outcome diagnosed since HIV seroconversion, regardless of subsequent diagnoses (termed “1^st^ outcome only”). This analysis eliminates confounding by the possibility that developing an initial disease may increase the risk of developing a second one. In the secondary analysis, individuals were included in a disease group if they had ever developed the disease (“anytime outcome”), regardless of whether it was the first or a subsequent disease diagnosis (thus, a single individual may be counted in multiple disease groups). This analysis is less rigorous than the first, but involves more events and was used to support or refute the primary analysis.

Of the six individual disease groups, significant (*p* <0.05) *KIR3DS1/Bw4-80I* protection against PCP, other OI, and MAC was observed in analyses considering 1^st^ outcome only, anytime outcome, or both (Table 1). Notably, there was no significant effect of this genotype against KS or AIDS lymphoma. These data indicated that *KIR3DS1/Bw4-80I* confers defense against opportunistic infections, but not against AIDS malignancies in general. Substantiating these findings, *KIR3DS1/Bw4-80I* was associated with strong protection against all OI outcomes combined (OR = 0.36–0.4, *p* = 0.003–0.005), but not against the combined AIDS malignancies (OR = 0.75–1.0, *p* = 0.5–0.9). Similar results were observed after increasing the control group from 88 to a total of 197 LTNP by the addition of 109 seroprevalent LTNP (those patients who were HIV+ at study entry, and remained free of AIDS for at least 13 y following entry into the study; for OI, OR = 0.4, *p* = 0.001; for malignancies, OR = 1.1, *p* = 0.8). The apparent lack of KIR3DS1/Bw4-80I protection against CMV retinitis, the only OI tested individually for which KIR3DS1/Bw4-80I showed no significant effect, could have to do with the immune privileged status of the retina where NK cell accessibility may be restricted regardless of the receptors they express. Indeed, there is little or no infiltration of inflammatory cells into the retina in association with CMV retinits [6].

The *Bw4-80I* group includes two alleles, B*57 and B*27, that are known to be highly protective in the acquired immune response against HIV based on both functional and genetic epidemiological data [7]. In contrast, B*35-Px associates with rapid progression to AIDS [7,8] and carries the Bw6 motif (i.e. not Bw4-80I). These three alleles show the strongest effects on progression to AIDS relative to all other individual *HLA* alleles in analyses of the cohorts used herein [7,9]. Thus, *KIR3DS1/Bw4-80I* protection could be artificially strengthened by the presence of the protective B*57 and B*27 in the *Bw4-80I* group and the absence of the susceptible B*35-Px from this group. However, removing patients with these alleles (*n* = 345) from the analysis did not alter the strength of *KIR3DS1/Bw4-80I* protection against OI (OR = 0.35, *p* = 0.019), indicating that the protection conferred by *KIR3DS1/Bw4-80I* is separate from the individual effects of these three alleles on the acquired immune response. Further, in a model adjusting for the effects of these three alleles as well as time to CD4 <200, the strength and significance of the *KIR3DS1/Bw4-80I* protection remained (OR = 0.34, *p* = 0.03). The lack of an effect of *KIR3DS1/Bw4-80I* on AIDS malignancy was not altered by removing or adjusting for these same variables.

The categorical analyses indicated that *KIR3DS1/Bw4-80I* protects against ever developing an OI, but not AIDS malignancy after HIV infection. Some patients with *KIR3DS1/Bw4-80I* do, however, develop OI even as a first AIDS outcome. We therefore tested whether *KIR3DS1/Bw4-80I* delayed progression to OI as a first AIDS outcome using 1,126 seroconverters in survival analysis (i.e. time since seroconversion to the first AIDS outcome diagnosed; Figure 1, Table 1). *KIR3DS1/Bw4-80I* was shown to delay progression to OI as a first outcome only [unadjusted: Relative Hazard (RH) = 0.56, *p* = 0.004; adjusted for time to CD4 <200 as a time-dependent covariate and HLA-B*57, -B*27, and -B*35-Px as fixed covariates in a Cox proportional hazards model: RH = 0.58, *p* = 0.007]. Further, delayed progression to OI was observed regardless of whether it was the first or a subsequent AIDS diagnosis (unadjusted: RH = 0.61, *p* = 0.008; adjusted: RH = 0.64, *p* = 0.016; unpublished data). This genotype showed no protection in survival analysis against progression to AIDS malignancy, whether it was the first outcome only or not (RH = 1.1–1.4, *p* = 0.3–0.8). Adjusting for race in the Cox model did not affect the results (RH = 1.34, *p* = 0.34 for malignancy; RH = 0.56, *p* = 0.004 for OI), which concurs with our previously observed effect of this compound genotype on AIDS progression in both European and African Americans [5]. Thus, the survival analysis confirmed and extended the observations of the categorical analyses with regard to both OI and AIDS malignancies.

**Figure 1:**
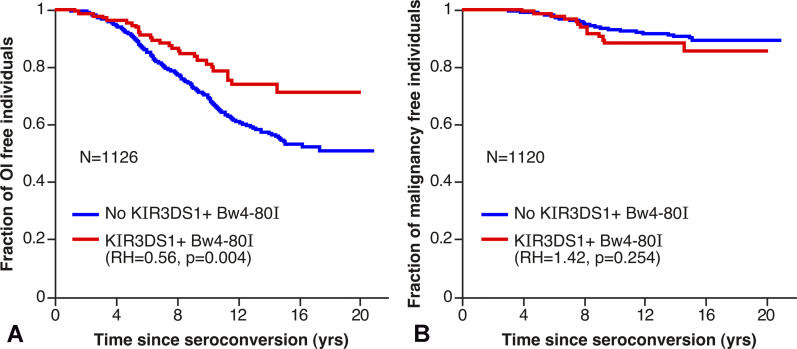
Effect of *KIR3DS1/Bw4-80I* on Progression to AIDS-Defining Illness Kaplan-Meier survival analyses illustrating the effect of *KIR3DS1/Bw4-80I* on progression to A) AIDS-defining opportunistic infections and B) AIDS-defining malignancies among seroconverters. Patients with *KIR3DS1/Bw4-80I* (red curve) were compared with patients missing this genotype (blue curve). RH and *p*-values from corresponding Cox models are given.

These data raised the possibility that the protective effect of *KIR3DS1/Bw4-80I* on AIDS progression that was reported previously may be completely due to protection against OI, rather than HIV directly. Viral load set point measurements (the mean of all viral load measurements determined between 6 mo to 3 y after infection) were available for 391 European American seroconverters from the MACS. The mean (natural log) viral load set point for patients with KIR3DS1/Bw4-80I (*n* = 47, mean = 9.4) was significantly lower than that for individuals without this genotype (*n* = 344, mean = 10.1; *p* = 0.01; Table 2), supporting a direct protective effect of KIR3DS1/Bw4-80I against HIV replication early after infection, and well before the development of AIDS-defining illnesses in all of the 391 patients studied here. This difference in viral load set points between the KIR3DS1/Bw4-80I positive vs. negative groups is likely to have clinical significance based on the correlation between viral load set point levels and time to AIDS that has been reported previously [10].

**Table 2:**
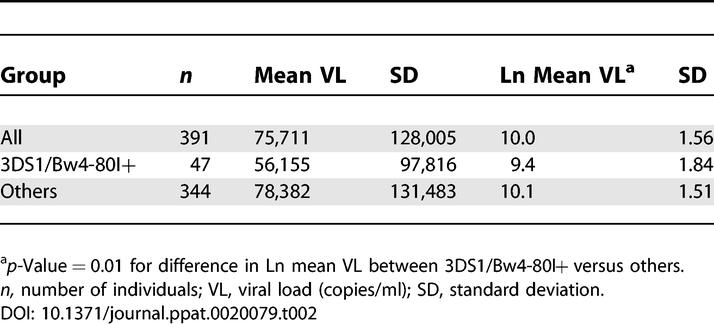
Summary of Mean HIV-1 RNA Load among 391 Seroconverters during the First 3 y after Seroconversion

These data indicate pleiotropic protective effects of *KIR3DS1/Bw4-80I* over the course of HIV disease, including relatively early control of viral load after infection and subsequently against developing OI (Figure 2). While these two protective effects are not completely independent of one another, adjusting for time to CD4 <200, a measurement linked to viral load set point [10], did not alter the protection conferred by *KIR3DS1/Bw4-80I* against OI. Notably, this genotype does not protect against AIDS malignancy. The conserved, activating NK cell receptor NKG2D has been implicated in surveillance against and elimination of tumor cells [11] and this receptor may also play a role in control of AIDS malignancies, beyond that of any activating KIR. To date, only a single genetic epidemiological study has implicated an activating *KIR* in tumor pathogenesis [12]. In this case, *KIR3DS1* was associated with increased risk of cervical neoplasia, possibly due to enhancement of chronic inflammation of the cervix, a likely precursor of cervical neoplasia [13]. Thus, environmental factors, variation in the HIV genome, or host genetic variants other than *KIR3DS1/Bw4-80I* may determine the risk of developing AIDS malignancies.

**Figure 2:**
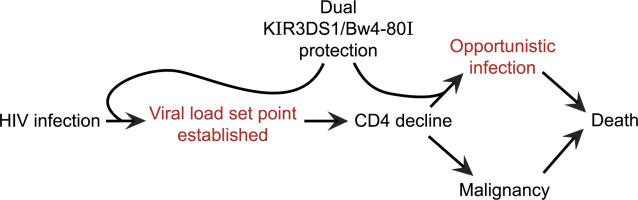
Bimodal Protection of *KIR3DS1*/*HLA-B Bw4-80I* in HIV-1 Infection Flow chart illustrating the dual protection conferred by *KIR3DS1*/*Bw4-80I* in the natural history of HIV-1 infection: early control of HIV-1 viral load, and late specific defense against opportunistic infections. There is no effect of this genotype on the development of AIDS-related malignancies.

It is not clear whether the protective effect of *KIR3DS1/Bw4-80I* is mediated through NK cells, CD8+ T cells, or both. NK cell activation through KIR3DS1 binding to ligand on HIV infected target cells may confer rapid protection against the virus very soon after infection and before the acquired immune response ensues. This does not preclude the possibility of KIR3DS1-mediated NK and/or T cell responses during chronic infection, however, and indeed the data presented here indicates protection against OI during the chronic phase of HIV infection. Functional studies will be necessary to define the stage(s) and effector cell type(s) responsible for the association of *KIR3DS1/Bw4-80I* with protection against AIDS that has been described previously [5] and herein.

The *HLA* class I loci show the strongest and most consistent effects (across studies) on HIV disease relative to any other single genetic locus. Of the three HLA class I loci, HLA-B is the most rapidly evolving [14] and it also appears to be the most consequential in the acquired immune response against HIV from both functional and genetic epidemiological perspectives [9,15]. On a population level, control of a rapidly evolving pathogen such as HIV likely requires host genetic factors that are highly polymorphic and evolving rapidly themselves, characteristics of both the *HLA* class I and *KIR* loci [9,15,16]. Thus, it may not be coincidental that allotypes of the *HLA-B* locus in combination with a *KIR* locus would be central to the innate immune response against both HIV and the opportunistic infections stemming from HIV-mediated immune suppression. It is now clear that *HLA* class I contributes heavily to both the acquired and the innate immune responses, and decoding the complexity of its effects in HIV disease may prove essential for conquering this virus.

## Materials and Methods

### Study participants.

Individuals infected with HIV-1 for whom dates of seroconversion were known were derived from four cohorts: the Multicenter AIDS Cohort Study (MACS) [17], the Multicenter Hemophilia Cohort Study (MHCS) [18], the San Francisco City Clinic Cohort (SFCCC) [19] and the AIDS Linked to Intravenous Experience (ALIVE) [20] (*N* = 1,184; European American = 799, African American = 332, Other = 53). There were several seroconverters for whom the date (after seroconversion) of first disease outcome was not known with regard to OI (*n* = 58) and malignancy (*n* = 64). Thus, we were unable to use these individuals in the survival analyses, but we were able to use them in categorical analyses, which do not take time to disease outcome after seroconversion into account. The categorical analyses employed a control group of 88 HIV+ LTNPs who had participated in their respective studies for at least 15 y and had never presented with disease symptoms (79 European Americans, five African Americans, five Other). Samples from long term nonprogressing seroprevalent individuals (individuals who entered the study HIV positive, were followed for at least 13 y, and never developed AIDS; *n* = 109) were used as additional controls in one set of categorical analyses in order to boost the sample size in the control group (99 European Americans, six African Americans, four Other). European American seroconverters with known viral load set point data were derived from the MACS (*N* = 391). This study was approved by the Protocol Review Office of the NCI institutional review board. Informed consent was obtained at the study sites from all individuals.

### *HLA* and *KIR* genotyping.

Genotyping of the *HLA* and *KIR* loci were performed as previously described [5].

### Viral load set point measurements.

Viral load measurements determined previously from 391 European American seroconverters were available for analysis from the MACS cohort. Prior to 1997, HIV-1 RNA copies/mL was measured retrospectively on stored samples using reverse-transcription polymerase chain reaction (Amplicor; Roche Diagnostics, Nutley, New Jersey) with an assay quantification limit of 400 copies/mL [10]. Samples below the quantification limit were subsequently tested with the Roche Ultrasensitive Assay with an assay detection limit of 50 copies/ml. Prospectively, HIV-1 RNA copies/mL are measured initially with Roche Ultrasensitive Assay whose detection limit is 50 copies/mL. All HIV RNA measurements obtained during the first 3 y after the first HIV-seropositive visit were used to calculate viral load set point values for each individual. Viral load measurements were first transformed to natural logarithm, and then averaged to determine the mean viral load set point for each individual. The average number of measurements per patient was 4.8 with a range of 1–10.

### Statistical methods.

Analyses were carried out on seroconverters from all of the cohorts combined. Although our previous study of *HLA* and *KIR* in these patients showed consistent effects in African Americans and European Americans [5], we performed the Cox model analysis adjusted and unadjusted for race. The two models yielded virtually identical results. Data management and statistical analyses were performed with SAS 9.1 (SAS Institute, Cary, North Carolina). PROC LOGISTIC was used for categorical analyses and PROC LIFETEST and PHREG were used for Kaplan-Meier and Cox model analyses. Statistical significance refers to two-sided *p*-values <0.05.

## Abbreviations

CMV: cytomegalovirus
CMVR: cytomegalovirus retinitis
HAART: highly active antiretroviral therapy
HLA: human leukocyte antigen
KIR: killer immunoglobulin-like receptor
KS: Kaposi sarcoma
LTNP: long-term nonprogressor
MAC: mycobacterium avium complex
MACS: the Multicenter AIDS Cohort Study
NK: natural killer
OI: opportunistic infection
PCP: pnuemocystis carinii pneumonia
RH: relative hazard
